# Emerging Roles of Ubiquitin-Specific Protease 25 in Diseases

**DOI:** 10.3389/fcell.2021.698751

**Published:** 2021-06-23

**Authors:** Wenjing Zhu, Dandan Zheng, Dandan Wang, Lehe Yang, Chengguang Zhao, Xiaoying Huang

**Affiliations:** ^1^Division of Pulmonary Medicine, The First Affiliated Hospital, Wenzhou Medical University, Wenzhou, China; ^2^School of Pharmaceutical Sciences, Wenzhou Medical University, Wenzhou, China

**Keywords:** USP25, deubiquitinating enzymes, antiviral immunity, neoplasm, Alzheimer disease

## Abstract

The balance of ubiquitination and deubiquitination plays diverse roles in regulating protein stability and cellular homeostasis. Deubiquitinating enzymes catalyze the hydrolysis and removal of ubiquitin chains from target proteins and play critical roles in various disease processes, including cancer, immune responses to viral infections and neurodegeneration. This article aims to summarize roles of the deubiquitinating enzyme ubiquitin-specific protease 25 (USP25) in disease onset and progression. Previous studies have focused on the role of USP25 in antiviral immunity and neurodegenerative diseases. Recently, however, as the structural similarities and differences between USP25 and its homolog USP28 have become clear, mechanisms of action of USP25 in cancer and other diseases have been gradually revealed.

## Introduction

Ubiquitination is a post-translational modification of proteins that is involved in regulation of the cell cycle and stress responses. It is the main signal that marks proteins for degradation by the ubiquitin-proteasome system (UPS). A protein that has been labeled with ubiquitin for degradation undergoes a series of enzymatic reactions and enters the 26S proteasome where it is degraded (Schwartz and Hochstrasser, 2003). Deubiquitination, which reverses ubiquitination, is mediated by deubiquitinating enzymes (DUB). These enzymes cleave the ubiquitin tag, which releases free ubiquitin for reuse and alters the fate of the substrate protein (Wing, 2003; Mevissen and Komander, 2017; Kaushal et al., 2018).

Analyses of the human genome have identified more than 100 functional DUBs, which have been classified into seven major families based on sequence and structural similarities: ubiquitin-specific proteases (USP), ubiquitin C-terminal hydrolases, ovarian tumor domain-containing proteases (OTU), the Machado-Joseph family of disease proteases (MJDs), the MIU-containing novel DUB (MINDY) family, Jad1/Pad/MPN domain-containing metalloenzymes (JAMM), and the newly discovered zinc fingers with UFM1-specific peptidase domain proteins/C6orf113/ZUP1 (ZUFSP)/Mug105 family (Mevissen and Komander, 2017; Haahr et al., 2018).

DUBs are capable of recognizing and cleaving peptide bonds or isopeptide bonds between ubiquitin and substrate or within the ubiquitin chain. DUBs remove a ubiquitin moiety, which is attached via its C-terminal glycine, from the nitrogen atom of a lysine or the N-terminal methionine of a substrate or another ubiquitin moiety (Basar et al., 2021). The catalytic structural domains of DUBs recognize specific positions or linkage types of ubiquitin chains; therefore, the process of deubiquitination is highly specific. Accordingly, deubiquitination is involved in regulating diverse and important cellular biological processes, including transcription, DNA damage repair and autophagy (Ramakrishna et al., 2011; Basar et al., 2021).

USPs represent the largest subfamily of DUB, and the roles for various members of this subfamily in human diseases have gradually been revealed. The imbalances in the ubiquitin system that result from alterations in USP function have been associated with several human diseases, including cancers and neurodegenerative diseases. Therefore, inhibitors against USP family DUBs are being developed to help further investigate biological mechanisms while also acting as the potential agents for the treatment of tumors and inflammation.

The importance of USP25 in particular continues to be uncovered, and recently, research has been performed to better understand the role of this enzyme. Therefore, this review focuses on the basic biology of USP25 and presents the latest discoveries about roles of USP25 in human diseases, such as infectious diseases, neurological diseases, and cancers.

## Molecular Characteristics of USP25

### Structure of USP25

USP25 was first identified by Valero et al. as the product of a gene on 21q11.2, one of the least densely populated regions of the human genome. The gene consists of 25 exons and encodes a 150 kDa protein consisting of 1087 amino acids (Valero et al., 1999). Three isoforms of USP25 are generated by alternative splicing. Isoform USP25a is found in most adult and fetal tissues. Isoform USP25b is found in all tissues except heart and skeletal muscle. Isoform USP25m is specifically present in the skeletal muscle and heart and is upregulated during myogenesis (Blount et al., 2012).

**FIGURE 1:**
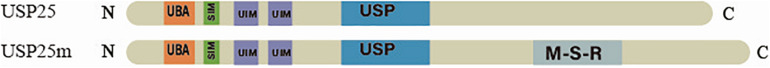
Structure of USP25. The structures of mammalian USP25 and its isoform USP25m. UBA, Ubiquitin-associated domain; SIM, SUMO-interacting motif; UIM, Ubiquitin-interacting motif; USP Domain, catalytic domain; M-S-R, Muscle-Specific Region.

Like most USPs, USP25 has three ubiquitin-binding domains (UBDs) at the N terminus: a ubiquitin-associated domain (UBA) and two tandem ubiquitin-interacting motifs (UIMs). However, no clear structural homology among USP family members has been detected in the extended C-terminal region outside of the catalytic domain (Denuc et al., 2009). Compared to the more common USP25a and USP25b isoforms, USP25m has an added muscle-specific domain (Figure 1).

**FIGURE 2:**
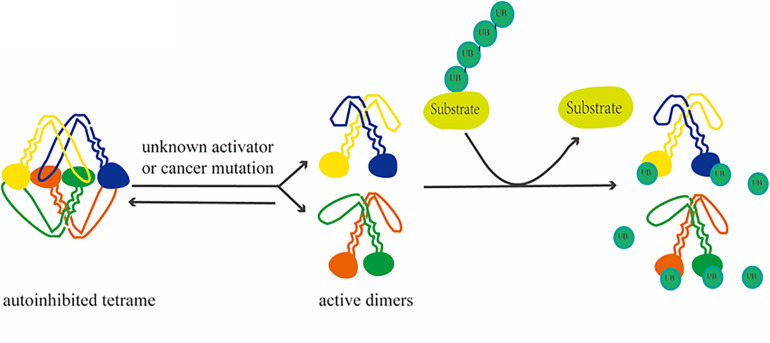
Depolymerization process of USP25. Activation of USP25, followed by activation from a self-inhibited tetramer to a dimer, removes ubiquitin from the substrate.

Tandem UIM regions, along with Lys48 binding, represent the active site in the linking of the ubiquitin chains together. Compared to enzymes in which a ubiquitin chain is attached at the Lys63 site, purified USP25 exhibited higher ubiquitin isopeptidase activity toward the Lys48 site, probably because the Lys48-linked ubiquitin chain substrate was able to be selectively held near the catalytic core (Kawaguchi et al., 2017). Meanwhile, USP25 is the first UIM protein identified to contain a small ubiquitin-like modifier (SUMO) interaction motif (SIM), which can bind to SUMO and promote covalent SUMO modification between UIMs, thus leading to the inhibition of deubiquitinating activity (Meulmeester et al., 2008) (Figure 2). Notably, USP25 is not only distributed in the nucleus and cytoplasm (Bosch-Comas et al., 2006; Denuc et al., 2009) but also can be localized to the endoplasmic reticulum (ER) (Blount et al., 2012), where it negatively regulates ER-associated degradation (ERAD).

### Regulation of USP25

Identification of the mechanisms governing the regulation of USP25 may be important to the development of appropriate therapeutic strategies. Information about the regulation of USP25 protein is limited, especially at the post-transcriptional level (Kessler and Edelmann, 2011), but currently known post-translational modifications of USP25 are shown in Table 1. Initial insight into regulatory mechanisms came when Cholay et al. (2010) showed that the second Src homology-2 domain (SH2) of spleen tyrosine kinase (SYK) physically interacted with a tyrosine-rich C-terminal region of USP25 when it is in the unphosphorylated state. Although the proteolytic activity of USP25 is not affected by subsequent SYK-mediated phosphorylation, the phosphorylation by SYK leads to changes in cellular levels of USP25 (Cholay et al., 2010).

**TABLE 1:** Regulator of USP25.

Regulator	Category	Mechanism	Function
SYK	Tyrosine kinase	Phosphorylates USP25	Regulates intracellular level of USP25
SUMO	Protein	Interacts with SUMO2/3	Sumoylates efficiently USP25
		Interacts with SUMO1	Sumoylates weakly USP25
Ubquitin	Protein	Conjugates to the lysine residue (Lys99)	Promotes USP25 catalytic activity
VRK2	Serine/threonine kinase	Phosphorylates Thr680, Thr727, and Ser745 residues of USP25	Impairs deubiquitination activity of USP25 and Upregulates TRiC protein
Smurf1	E3 ligase	Affects the stability of USP25 by modifying its Lys48-linked ubiquitination.	Degrades USP25
MiR-27a-3p	miRNA	Downregulates transcription of USP25	Inhibits migration and invasion of trophoblast

*A summary of the modification of USP25.*

Later, Kim et al. (2015) showed that phosphorylation of USP25 by vaccinia-associated kinase 2 (VRK2) may be a critical step in the pathway leading to destabilization of tailless complex polypeptide 1 ring complex (TRiC) proteins, which have molecular chaperone activity. USP25 interacts with and deubiquitinates TRiC, which protects it and ultimately leads to a reduction in the accumulation of misfolded polyglutamine protein aggregates. Upon VRK2-mediated phosphorylation of Thr680, Thr727 and Ser745 of USP25, the deubiquitination activity of USP25 is inhibited, resulting in ubiquitination and destabilization of TRiC (Kim et al., 2015).

USP25 function can be regulated by other post-translational modifications in addition to phosphorylation. For instance, SMAD specific E3 ubiquitin protein ligase 1 (SMURF1) affects the stability of USP25 by modifying its Lys48-linked ubiquitination. This mechanism impacts infectious disease propagation in that SMURF1 has been shown to limit antiviral functions of USP25 by promoting its ubiquitination and degradation (Qian et al., 2018). Specifically, the USP25m isoform can be monoubiquitinated and is capable of auto-deubiquitination. This ubiquitination interacts with another modification, addition of small ubiquitin-like modifier (SUMO). UBDs of USP25m facilitate monoubiquitination at the preferred site, Lys99, which has previously been shown to be a target of sumoylation, which inhibits USP25 enzymatic activity. The mutation of Lys99 significantly attenuates deubiquitination of myosin binding protein C1 (MyBPC1) by USP25m. Thus, USP25m is regulated by the alternative conjugation of ubiquitin (activation) or SUMO (inhibition) to Lys99, which may facilitate interactions with other intramolecular regulatory domains (Denuc et al., 2009).

Furthermore, Mohideen et al. elucidated the existence of an interaction between USP25 and SUMO2/3 proteins (Mohideen and Lima, 2008). While USP25 is only weakly sumoylated with SUMO1, its modification with SUMO2/3 is dependent on a SIM within USP25. SUMO2/3-specific binding and conjugation can occur at seven amino acids in the SIM of USP25. Sumoylation and efficient hydrolysis of the ubiquitin chain occur within overlapping UIMs of USP25; thus, the binding and hydrolysis of the ubiquitin chain are affected by sumoylation (Meulmeester et al., 2008). The findings of Yang et al. also confirmed that the catalytic activity of USP25 was inhibited when SUMO2 was non-covalently bound to the USP25 SIM, as SUMO2 can competitively block the interaction between USP25 and its ubiquitinated substrate (Yang et al., 2017). On the other hand, Guzzo et al. also found that tandem SIM-UIMs could increase the possible linkages of SUMO-ubiquitin hybrid chains. In turn, SUMO and ubiquitin-mediated signaling can be amplified through the interaction of hybrid SUMO–ubiquitin chains and their receptors (Guzzo and Matunis, 2013).

Similarity analyses among evolutionarily related proteins has revealed additional potential regulatory mechanisms. Based on genomic and protein sequences and functional data, USP25 has been shown to be homologous to USP28 (Valero et al., 2001). The comparison of the molecular characteristics of the two revealed that USP28 is a constitutively active dimer. In contrast, the crystal structure of USP25 shows an unexpected combination in which a homotetramer is formed by the union of two dimers. This quaternary structure leads to a unique autoinhibitory mechanism in which the autoinhibitory motif of USP25 directly occupies a large portion of the ubiquitin-binding surface, ultimately leading to the inhibition of its enzymatic activity (Sauer et al., 2019). In full-length enzymes, the C-terminal domain does not affect oligomerization, but the N-terminal region affects the dimer–tetramer equilibrium *in vitro* (Gersch et al., 2019). Furthermore, the activation of USP25 *in vitro* and *in vivo* is achieved through mutations associated with cancer, suggesting a functional link between the auto-inhibitory and pro-cancer effects (Sauer et al., 2019).

The activity of USP25 is potentially regulated at the transcriptional level as well as at the post-translational level. Transcription of USP25 has been shown to be down regulated by miR-27a-3p. This effect contributes to the epithelial-mesenchymal transition (EMT) process and thus inhibition of trophoblast migration and invasion (Ding et al., 2019). Insufficient trophoblast invasion capacity may be associated with the occurrence of recurrent miscarriage (Ding et al., 2019).

## Effect of USP25 in Diseases

### Functions of USP25 in Alzheimer’s Disease

The direct involvement of ubiquitinated protein aggregates in pathological conditions has not yet been demonstrated. However, the accumulation of ubiquitin conjugates has been described in patients with Alzheimer’s disease, Parkinson’s disease, and Huntington’s disease (Valero et al., 2001). Down syndrome, which is caused by trisomy of chromosome 21, is an important risk factor for early-onset Alzheimer’s disease. Zheng et al. constructed a combined mouse Down syndrome/Alzheimer’s disease model and found that a triploid combination model of the homologous chromosome 21 gene exacerbated neuroinflammation. The overexpression of the deubiquitinating enzyme USP25, which is encoded by a gene located on chromosome 21, led to microglial activation and induced synaptic and cognitive dysfunction. However, ablation of the USP25 gene reduced neuroinflammation and rescued synaptic and cognitive function in a mouse model. Mechanistically, the deletion of USP25 attenuated microglia-mediated overproduction of pro-inflammatory cytokines. The inhibition of USP25 re-established homeostatic microglial signaling and restored synaptic and cognitive function in mice (Zheng et al., 2021).

ERAD is a coordinated action in which misfolded or abnormal proteins are identified, ubiquitinated and extracted from the ER, and ultimately sent to the proteasome for degradation. Blount et al. proposed that USP25 counteracted the ubiquitination of ERAD substrates that had been mediated by the ERAD marker synoviolin, an E3 ubiquitin ligase. This action of USP25 would divert substrates away from proteasomal degradation (Blount et al., 2012). In addition, Jung et al. found that decreased levels of USP25 and increased levels of synoviolin were also observed during ER stress. An important implication of these changes is an overall destabilization of amyloid precursor protein (APP) in Alzheimer’s disease pathogenesis. APP is rapidly degraded by UPS in response to ER stress, leading to the formation of β-amyloid (Aβ), the main pathological marker of Alzheimer’s disease. USP25 promotes the stability of full-length APP by interacting with APP, leading to the accumulation of misfolded APP (Jung et al., 2015).

*In silico* analyses of miRNA target prediction unveiled that miR-455-3p had a strong capacity to bind to USP25, and human miRNA-455-3p thus might serve as a peripheral biomarker of Alzheimer’s disease. Kumar and Reddy further validated the high expression of miR-455-3p in fibroblasts and B lymphocytes from patients with familial and sporadic Alzheimer’s disease obtained from the Coriell Cell Bank of the National Institute on Aging Differences and in postmortem analyses of brains obtained from the National Institutes of Health NeuroBioBank (Kumar and Reddy, 2018). In short, USP25 plays a promoting role in the development of Alzheimer’s disease.

### Functions of USP25 in Antiviral Immunity

**FIGURE 3:**
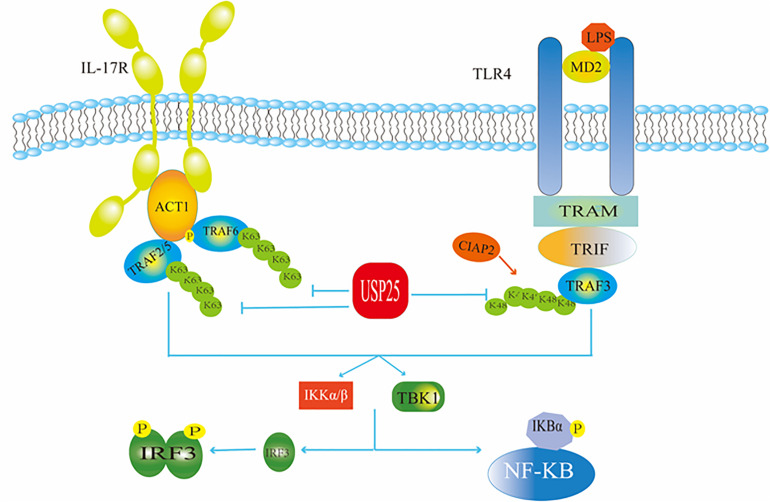
USP25 in antiviral immunity. USP25 affects the IRF and NF-κB pathways by acting on ubiquitination of TRAF in the IL-17R and TLR4 pathways, respectively, thereby regulating the downstream molecules.

USP25 negatively regulates signaling triggered by interleukin 17 (IL-17), a mediator of inflammation that plays a critical role in autoimmunity and infection (Zhong et al., 2012; Figure 3). For example, Zhong et al. found that the overexpression of USP25 inhibited IL-17-triggered signaling and inflammation by interacting with tumor necrosis factor receptor associated factor 5 (TRAF5) and TRAF6. USP25 was required for the deubiquitination of TRAF5 and TRAF6, but not of other TRAF proteins. Mechanistically, IL-17 stimulation induced the association of USP25 with TRAF5 and TRAF6, and the USP25 deubiquitination activity opposed the activity of the Act1 E3 ubiquitin ligase, which ubiquitinates Lys63 in both TRAF5 and TRAF6. In addition, USP25 is involved in the processes by which toll-like receptors (TLR) monitor and recognize a variety of different disease-associated molecular patterns and provide the first barrier of the body against infectious diseases. Among these receptors is TLR4, which recognizes Gram-negative bacterial lipopolysaccharides (LPS). USP25 deubiquitinates the adaptor protein TRAF3 to regulate TLR4-dependent innate immune responses (Figure 3). Upon stimulation of TLR4 by LPS, USP25 was shown to specifically reverse the Lys48-linked ubiquitination of TRAF3 mediated by the E3 ubiquitin ligase inhibitor of apoptosis 2 (cIAP2). This deubiquitination enhanced the activation of the transcription factor interferon regulatory factor 3 (IRF3) but attenuated TLR4-induced activation of nuclear factor kappa-B (NF-κB) (Zhong et al., 2013). NF-κB is a nuclear transcription factor whose activation is required to enter the nucleus to affect gene transcription and expression. When the body is in a normal state, the NF-κB dimer binds to its inhibitor κB (IκB), which exists as an inactive trimer in the cytoplasm. After a viral invasion, IκB is degraded, and its inhibitory effect on NF-κB is lost (Colleran et al., 2013). IRF3 is also a nuclear transcription factor that exists in the cytoplasm when the cells are not stimulated. When a virus invades a cell, it can react with a corresponding receptor to induce IRF3 phosphorylation and activation through the formation of homodimers (Kawai and Akira, 2008). The TIR-domain-containing adapter-inducing interferon-β (TRIF)-dependent pathway is initiated by toll-like receptor adaptor molecule 1 (TRAM) and TRIF targeting some specific TLRs. TRIF is essential for the recruitment of TRAF3. Subsequently, TRAF3 recruits the inhibitor of NF-κB kinase (IKK)-related kinases, and TRAFKC family member associated NF-κB activators bind to the tank-binding kinase (TBK1), leading to the phosphorylation of IRF3 and forming a dimer. This leads to translocation to the nucleus and results in the activation of IFN-inducible genes and inflammatory mediator expression (Mohammad and Thiemermann, 2020).

Lin et al. further found that compared with wild-type mice, USP25-deficient mice were more susceptible to infection with herpes simplex virus 1 or influenza A virus subtype H5N1. Upon infection with an RNA or DNA virus, USP25 associates with TRAF3 and TRAF6 and protects TRAF3 and TRAF6 from virus-induced proteasome-dependent or independent degradation (Lin et al., 2015). Similarly, Ren et al. found that upregulation of USP25 induced by Sendai virus was attenuated in cells lacking IRF7. Sendai virus or type I interferon-induced USP25 upregulation required *de novo* protein synthesis of IRF7. In addition, IRF7 bound directly to two conserved IRF-binding sites on the USP25 promoter to drive USP25 transcription (Ren et al., 2016).

Another infection-related function of USP25 involves the degradation of TRAF3, which induces endotoxin tolerance in macrophages. Wen et al. found that knockdown of USP25 correlates with increased Lys48-linked ubiquitination of TRAF3, thereby inducing endotoxin tolerance in endotoxin-tolerant Kupffer cells, while overexpression of USP25 attenuated these pro-inflammatory effects of the USP25 knockdown (Wen et al., 2019). Long et al. additionally reported that the endotoxin LPS elevated the binding of histone acetyltransferase to origin recognition complex 1 (HBO1) protein levels by reversing HBO1 ubiquitination through the upregulation of USP25 and that this effect altered inflammatory gene transcription in Tohoku Hospital Pediatrics-1 (THP-1) monocytes and human primary macrophages (Long et al., 2018).

### Functions of USP25 in Cancer

Disruptions of the proteasome system via alterations of USP25 activity have been associated with various human cancers (Table 2). For example, Deng et al. found that USP25 was overexpressed more than threefold in breast cancer tissues compared with adjacent normal tissues, indicating a significantly enhanced role of the UPS in breast cancer (Deng et al., 2007). A similar result was found in hepatocellular carcinoma (HCC). In this regard, Fujimoto et al. sequenced and analyzed the whole genomes of 27 HCC samples. The results indicated that USP25 was a commonly found mutation. Its specific role, then, in HCC deserves further investigation (Fujimoto et al., 2012).

**TABLE 2:** Function of USP25 in cancer.

Cancer type	Cell lines	Mechanism/pathway	References
**Experimental evidence**			
prostate cancer	PC-3 and LNCaP	Selective inhibition-C44 disrupts the interaction between TNKS and USP25, leading to a higher half-life of AXIN, which in turn regulated the Wnt/β-catenin pathway.	Cheng et al., 2019
colorectal cancer	Ls174T and HCT116 cells	Vismodegib suppresses the expression levels of its related substrate proteins, including c-Myc, Notch1 and tankyrase-1/2 *in vitro* and in colorectal cell lines.	Wang et al., 2020a
non-small-cell lung cancer (NSCLC).	A549, H1299, SPC-A-1sci, SPC-A-1, XL-2, H460, H358, and HEK-293T	MiR-200c negatively regulates migration and invasion abilities by targeting USP25.	Li et al., 2014
colorectal cancer	DLD-1, SW480, and HCT116	Deficiency of USP25 promotes the degradation of tankyrases and consequently leads to the stabilization of Axin to negatively regulate Wnt/β-catenin signaling. The C terminus of USP25 directly interacted with TNKS1.	Xu et al., 2017
EGFR-dependent tumors	HeLa cells	USP25 restrains the degradation of the EGFR by promoting the association of the E3 ubiquitin ligase c-Cbl with EGFR in the early steps of EGFR internalization.	Niño et al., 2020
Ph-positive leukemia	Human CML cells (K562 and KU812) acute lymphoblastic leukemia cells (SK-9 and MOLT-4) T cell leukemia Jurkat	The depletion of USP25 mediated by shRNA increases ubiquitinated BCR-ABL, which can promote the degradation of BCR-ABL protein in Philadelphia (Ph)-positive leukemia cells.	Shibata et al., 2020
colorectal cancer	Mouse model	Knockout or pharmacologic inhibition of USP25 attenuated Wnt and SOCS3–pSTAT3 signaling to inhibit colonic tumorigenesis.	Wang et al., 2020b
**Data analysis**			
breast cancer		USP25 is over-expressed (>threefold) in breast cancer tissue compared to adjacent normal breast tissue.	Deng et al., 2007
HCC		Significantly frequent mutation	Fujimoto et al., 2012
stomach adenocarcinoma		Overexpression of USP25 indicated the poor outcome	Fang and Lu, 2020
large cell lung carcinoma		Putative tumor suppressor gene	Yamada et al., 2008
lung adenocarcinoma		Potential target	Yin and Yu, 2019

*The roles and pathways of USP25 in regulating the development of various cancers.*

The combination of bioinformatic tools, including sequencing, and data analysis of experimental results is widely used by an increasing number of research teams to find potential clinical targets associated with tumors. Based on the analysis of available databases, the overexpression of USP25 was found to indicate a poor outcome in stomach adenocarcinoma (Fang and Lu, 2020). Yamada et al. analyzed the presence of putative tumor suppressor genes in the relevant genomic region due to the frequent loss of heterozygosity at 21q21 in lung cancer and the identification of homozygous deletions in this region. Here, USP25 was found to be frequently downregulated and was located in regions commonly deleted in human lung cancer (Yamada et al., 2008).

In experimental analyses, Li et al. found that miR-200c exerted tumor-suppressive effects on non-small cell lung cancer (NSCLC) by inhibiting the expression of USP25. MiR-200c reduced the mRNA and protein levels of USP25 by directly binding to the 3′-untranslated region of the USP25 mRNA. These results were corroborated by an analysis of clinical data, which showed that USP25 protein and mRNA levels were higher in patients with NSCLC compared with healthy controls and that they correlated with clinical stage and lymph node metastasis (Li et al., 2014). Moreover, lung cancer cells in which USP25 was knocked down had diminished migration and invasion abilities (Yin and Yu, 2019). USP25 has been shown to be a potential target for a specific treatment: the use of hematoporphyrin derivatives (HPD) in the treatment of lung adenocarcinoma. HPD has a sensitizing effect on lung adenocarcinoma. Yin and Yu performed RNA sequencing on the lung adenocarcinoma cell line A549 following mock treatment or treatment with x-ray radiation or x-ray radiation and HPD. The targeting of USP25 by miR-200c in this system was demonstrated after network integration of 464 differentially expressed genes from the sets of genes with significantly altered expression between the x-ray and HPD-treated and untreated or x-ray-treated groups. Further support for USP25 as a target of HPD came with the identification that USP25 was also influenced by miR-200b and miR-429 (Yin and Yu, 2019).

In addition, Xu et al. found that USP25 positively regulated Wnt/β-catenin signaling. Wnt signaling is negatively regulated by the scaffolding protein axin, which controls the rate-limiting step in β-catenin disruption. In Wnt-stimulated cells, axin is rapidly modified by tankyrase-mediated poly(ADP-ribosyl)ation, which promotes axin proteolysis. The team found that the deletion of USP25 promoted the degradation of tankyrases, which in turn stabilized axin to antagonize Wnt signaling. The interaction between tankyrases 1 (TNKS1) and USP25 was further characterized by X-ray crystal structure determination (Xu et al., 2017). Based on these results, Cheng et al. investigated the USP25/TNKS-ARC5 protein complex by performing a hierarchical virtual screening of more than 200 000 compounds in the characterized structure. The analysis led to the discovery that the small molecule C44 disrupted the interaction between TNKS and USP25, leading to an increase in the half-life of axin, which in turn regulated the Wnt/β-catenin pathway. In addition, *in vivo* and *in vitro* experiments revealed that the selective inhibition of TNKS–USP25 interaction by C44 significantly reduced prostate cancer cell proliferation (Cheng et al., 2019).

An important mechanism by which cancer cells present uncontrolled epidermal growth factor receptor (EGFR) signaling is the evasion of receptor downregulation. The ubiquitination of EGFR plays a decisive role in this process because it regulates receptor internalization, translocation, and degradation. Niño et al. used qualitative and quantitative assays to establish USP25 as a negative regulator of the EGFR downregulation process to impede oncogenic growth signaling in EGFR-dependent tumors. In the early steps of EGFR internalization, USP25 could inhibit EGFR degradation by promoting the association of the E3 ubiquitin ligase c-Cbl with EGFR (Niño et al., 2020).

Fusion genes resulting from chromosomal rearrangements are frequently found in various cancer cells, such as the breakpoint cluster region (BCR)-c-abl oncogene 1 (ABL) in chronic myeloid leukemia (CML). The depletion of USP25 mediated by shRNA increases ubiquitinated BCR-ABL, which can promote the degradation of BCR-ABL protein in Philadelphia (Ph)-positive leukemia cells. These investigations are particularly important, because resistance to tyrosine kinase inhibitors is not uncommon in the treatment of patients with CML. The team found that pharmacological inhibition of USP25 induced rapid degradation of BCR-ABL protein in cells carrying the Philadelphia chromosome, regardless of the patient’s sensitivity to tyrosine kinase inhibitors. Thus, this may be an effective way to overcome kinase inhibitor resistance (Shibata et al., 2020).

Accumulating evidence suggests the critical roles of gut bacteria in colorectal cancer development by directly activating protumor factors or indirectly providing toxic factors (Wang et al., 2020b). The knockout or pharmacological inhibition of USP25 after induction of experimental bacterial infection or colitis was investigated by Wang et al. (2020b). The team concluded that USP25 not only promoted the clearance of infected bacteria and enhanced the immune response and resolution of inflammation, but also upregulated Wnt and suppressor of cytokine signaling 3-phosphorylated signal transducer and activator of transcription 3 signaling, thereby inhibiting colon tumorigenesis (Wang et al., 2020b).

### Effects of USP25 in Other Diseases

While USP25 has important roles in cancer, it similarly plays a critical part in other diseases, including diabetes and metabolic diseases of muscle and adipocytes. The key to systemic glucose homeostasis is the ability of adipocytes and muscle cells to sequester the glucose transporter type 4 (GLUT4) in intracellular compartments. Further studies on USP25, therefore, were conducted in adipocytes (Habtemichael et al., 2018; Sadler et al., 2019). USP25m is also expressed in adipocytes, and insulin mediates cleavage of the tether containing UBX domain of GLUT4 (TUG) via USP25m, and this cleavage liberates GLUT4 storage vesicles from the Golgi and activates their microtubule-based movement to the plasma membrane (Sadler et al., 2019). The muscle isoform USP25m specifically interacts with MyBPC1 and rescues MyBPC1 from proteasomal degradation to maintain stability and increase its cellular half-life (Gersch et al., 2019). USP25m also interacts with two other sarcomeric proteins, actin α-1 (ACTA1) and filamin C (FLNC), which are two of the three sarcomeric proteins that are essential for muscle maintenance and differentiation and are implicated in the pathogenesis of severe myopathies (Gersch et al., 2019).

In relation to lung disease, Long et al. found that components of cigarette smoke increased bacterial load in lung epithelial cells via downregulation of USP25, which catalyzed deubiquitination of histone deacetylase 11 (HDAC11) and regulated HDAC11 protein stability. They assumed that the down-regulated USP25/HDAC11 axis mediated by CSE might contribute to the high susceptibility of the smoking population to bacterial infections (Long et al., 2020).

## Development of Inhibitors of USP25

As demonstrated, the USP25 enzyme is associated with many diseases, so it is necessary to develop compounds that inhibit its catalytic activity. To date, researchers have developed inhibitors that target not only USP25, but also its closest homolog, USP28. For example, the FDA-approved drug vismodegib, originally used to treat basal cell carcinoma by inhibiting the Hedgehog (Hh) signaling pathway has been shown to be an inhibitor of USP25. Wang et al. found that this inhibition suppressed the expression levels of its related substrate proteins, including c-Myc, Notch1 and tankyrase-1/2 *in vitro* and in colorectal cell lines. The binding pocket for vismodegib in USP25 is within two helices that span Asp262 to Glu285 and Asn293 to Tyr300 (Wang et al., 2020a).

In addition, Wrigley et al. revealed that another set of compounds, AZ1 through AZ4, were able to non-competitively inhibit USP25 and USP28 (Wrigley et al., 2017). Accordingly, Wang et al. found that in a mouse model, AZ1 was able to impair USP25-induced bacterial infection in the intestine and to enhance the immune response, while inhibiting the role of USP25 in the promotion of intestinal cancer (Wang et al., 2020b). Wrigley et al. demonstrated that these inhibitors can trigger modulation of the half-life and total levels of c-Myc in cells and can induce loss of cell viability and apoptosis in a panel of cancer cell lines (Wrigley et al., 2017). Clearly, it is of interest that these inhibitors are used as chemical probes to study the biological properties and functions of USP28. Unfortunately, since USP25 and USP28 sequences are highly similar, it will likely be challenging to develop selective inhibitors of USP25.

## Conclusions and Perspectives

Deubiquitinating enzymes maintain protein turnover, signaling, and response to various stresses by removing ubiquitin modifications from substrate proteins. In some cases, studies are limited to only the analysis of public data. Hence, more corresponding trials should be performed to investigate and explore the underlying mechanisms more deeply and comprehensively. Regulation of USP25 has diverse physiological and pathological effects; therefore, more regulators and targeting substrates of USP25 need to be discovered. An increasing number of deubiquitinating enzymes are being used as the targets for therapeutic applications. USP25 tends to play a facilitating role in tumor development, but many of the specific mechanisms are not fully detailed, and inhibitors of USP25 have not yet been broadly studied in tumor therapy. Of course, the therapeutic implications of USP25 should not be overlooked as well; inhibitors may, for example, serve to enhance the sensitivity of tumors to multiple treatment strategies, including radiation therapy, traditional chemotherapy, immunotherapy, and molecular-targeted therapy. Importantly, it should be remembered that the therapeutic role of USP25 in diseases is not limited to tumors.

## Author Contributions

WZ and DZ collected and analyzed the literature and wrote the manuscript. DW provided intellectual input and analyzed the literature. LY, CZ, and XH supervised the review process. All authors have read and approved the final manuscript.

## Conflict of Interest

The authors declare that the research was conducted in the absence of any commercial or financial relationships that could be construed as a potential conflict of interest.
